# Thyroid Nodule Size and Prediction of Cancer: A Study at Tertiary Care Hospital in Saudi Arabia

**DOI:** 10.7759/cureus.7478

**Published:** 2020-03-30

**Authors:** Hadi Afandi Al-Hakami, Raneem Alqahtani, Asim Alahmadi, Dakheelallah Almutairi, Mohammed Algarni, Talal Alandejani

**Affiliations:** 1 Otolaryngology, King Saud bin Abdulaziz University for Health Sciences, King Abdullah International Medical Research Center, Ministry of National Guard Health Affairs, Jeddah, SAU; 2 Medicine, College of Medicine, King Saud Bin Abdulaziz University for Health Sciences, Jeddah, SAU; 3 Otolaryngology, Ohud Hospital, Madinah, SAU; 4 Otolaryngology, King Saud Bin Abdulaziz University for Health Sciences, King Abdullah International Medical Research Center, King Abdulaziz Medical City, Ministry of National Guard Health Affairs, Jeddah, SAU; 5 Otolaryngology, King Saud Bin Abdulaziz University for Health Sciences, King Abdulaziz Medical City, Jeddah, SAU; 6 Surgery, King Abdulaziz Medical City, Ministry of National Guard Health Affairs, Jeddah, SAU; 7 Otolaryngology, King Saud Bin Abdulaziz University for Health Sciences, Jeddah, SAU

**Keywords:** thyroid nodule, malignancy risk, thyroid malignancy, thyroid, thyroid nodule size, fine-needle aspiration

## Abstract

Background

Thyroid noduleshave become relatively common in clinical practice,and their prevalence increases with age. The majority of thyroid nodules are benign, with 5-15% being malignant. There are a number of well-established predictors of malignancy in thyroid nodules, but thyroid nodule size has been a cause for concern for many researchers and results of the studies are still controversial about their probability of malignancy. Up to the current knowledge, there is no published study that evaluates if thyroid nodule size is associated with the risk of malignancy in Saudi Arabia, so in this study, we aim to find that.

Methods

This is a retrospective study of 987 patients who underwent thyroid nodule fine-needle aspiration (FNA) and subsequent thyroidectomy for thyroid nodules measuring ≥ 1 cm.

Results

Thyroid cancer was more prevalent in males than females, and in patients who were older than or equal to 45 years. Nodular size of 1 - 1.9 cm was more prevalent among cancer patients than in benign cases (p<0.001).

Conclusions

The highest malignancy risk was observed in nodules <2 cm and no increase in malignancy risk for nodules >2 cm. Nevertheless, when examined by type of thyroid malignancy, the rate of follicular carcinoma and other rare malignancy increased with increasing nodule size.

## Introduction

Thyroid nodules have become relatively common in clinical practice, and their prevalence increases with age. Before the introduction of routine imaging, thyroid nodules were found in approximately 5-10% of people by palpation alone [1]. The prevalence of thyroid nodules varies depending on age, gender, and geographic location, and now is estimated to be 20% to 60% [2, 3]. The majority of thyroid nodules are benign, with 5% to 15% being malignant [1]. Moreover, the worldwide incidence of thyroid cancer has been increasing over the past decade, due to the diagnosis of asymptomatic disease secondary to the advancement and improvement of diagnostic technology and increased surveillance [4]. The initial screening and triage of thyroid nodules are carried out using the fine needle aspiration (FNA), which has been broadly accepted as the most cost-effective and accurate tool [5]. Given that 70% of aspirates return benign, FNA has proven to be of high value. However, 20-25% of samples return indeterminate, making FNA imperfect [6]. Prediction of thyroid malignancy is assisted by investigation of various clinical and radiological risk factors, including increased nodule height versus width, microcalcifications on ultrasonography, prior ionizing radiation exposure, nodule fixation, and firmness, hoarseness of voice, and age [1]. The nodule size has been a cause of concern for many researchers; some studies have reported an increased risk of malignancy with increasing nodule size, while many others denied an independent relationship. Nevertheless, data on the size of thyroid nodules as a determinant of carcinoma are still controversial. Different researches have been conducted for different aims. However, given the lack of published studies in Saudi Arabia, we aim to determine the impact of nodule size on the risk of thyroid cancer.

## Materials and methods

After obtaining Institutional Review Board approval from the Regional Research and Ethics Committee at King Abdullah International Medical Research Center Western Region, we performed a retrospective review of 987 patients who presented with a thyroid nodule (≥ 1 cm), underwent thyroid nodule FNA and subsequent thyroidectomy by surgeons in the Division of Otolaryngology - Head and Neck Surgery from January 2010 to December 2018 at King Abdulaziz Medical City (KAMC) in Jeddah, Western Region, Saudi Arabia. A self-developed data collection sheet was created to collect and organize the recruited data. It included different items such as patient demographics, family history of thyroid cancer, number of nodules, nodule size by ultrasound, thyroid-stimulating hormone (TSH) level, results of FNA, type of surgical operation, as well as final histopathologic diagnosis. Fine needle aspiration biopsy (FNAB) results were categorized according to Bethesda classification as non-diagnostic (category I), benign (category II), atypia of undetermined significance (AUS)/ follicular lesion of undetermined significance (FLUS) (category III), follicular neoplasm/suspicious for a follicular neoplasm (category IV), suspicious for malignancy (category V), and malignancy (category VI) groups. 

For data entry, Microsoft Office Excel (Microsoft Corporation, Redmond, US) was used, and Statistical Package Social Sciences (SPSS; IBM Inc., Armonk, US) software version 25.0 for data analysis. For statistical analysis, descriptive statistics and Pearson's chi-squared (χ2) test were used, and missing data were excluded. Statistical significance was defined as a p-value of <0.05.

## Results

A total of 987 patients presented with thyroid nodule (≥ 1 cm) underwent thyroid nodule FNA and subsequent thyroidectomy during the study period. As shown in Table 1, the studied patients comprised of 689 females (69.8%) and 298 males (30.2%). Their age ranged between 18-80 years, and (62%) were aged 45 years and above. Five hundred sixty-seven patients (57.4%) had multiple nodules, and 420 patients (42.6%) had solitary nodules. Thyroid function was normal in almost all patients (93.9%), and 270 patients (27.4%) gave a positive family history of thyroid cancer. Nodules' size was equal or greater to 2 cm in 70.5% of the patients. Malignant nodules were found in 30.5% of the studied cases. Total thyroidectomy was found to be the most applied surgical operation on the sample cases occupying 65.5%, followed by hemithyroidectomy - 34.5%. 

**Table 1:** Demographic data of the patients SD - standard deviation; FNA - fine needle aspiration; AUS - atypia of undetermined significance; FLUS - follicular lesion of undetermined significance

Characteristics	Patients (n=987) [n (%)]
Age (years)	
<45	375 (38)
≥45	612 (62)
Mean ± SD	48.36 ±13.03
Range	18-80
Gender	
Females	689 (69.8)
Males	298 (30.2)
Number of nodules	
Solitary	420 (42.6)
Multiple	567 (57.4)
Family history of thyroid cancer	
Yes	270 (27.4)
No	717 (72.6)
Thyroid function	
Hypothyroid	16 (1.6)
Euthyroid	927 (93.9)
Hyperthyroid	44 (4.5)
Distribution of FNA cytology	
Nondiagnostic or unsatisfactory	71 (7.2)
Benign	355 (36)
AUS/FLUS	180 (18.2)
Suspicious for a follicular neoplasm	118 (12)
Suspicious for Malignancy	135 (13.7)
Malignant	128 (13)
Diagnosis	
Benign	686 (69.5)
Malignant	301 (30.5)
Size of nodules (cm)	
1.0‑1.9	291 (29.5)
2.0‑2.9	319 (32.3)
3.0‑3.9	214 (21.7)
≥4	163 (16.5)
Type of operation	
Hemi thyroidectomy	341 (34.5)
Total thyroidectomy	646 (65.5)

**Figure 1:**
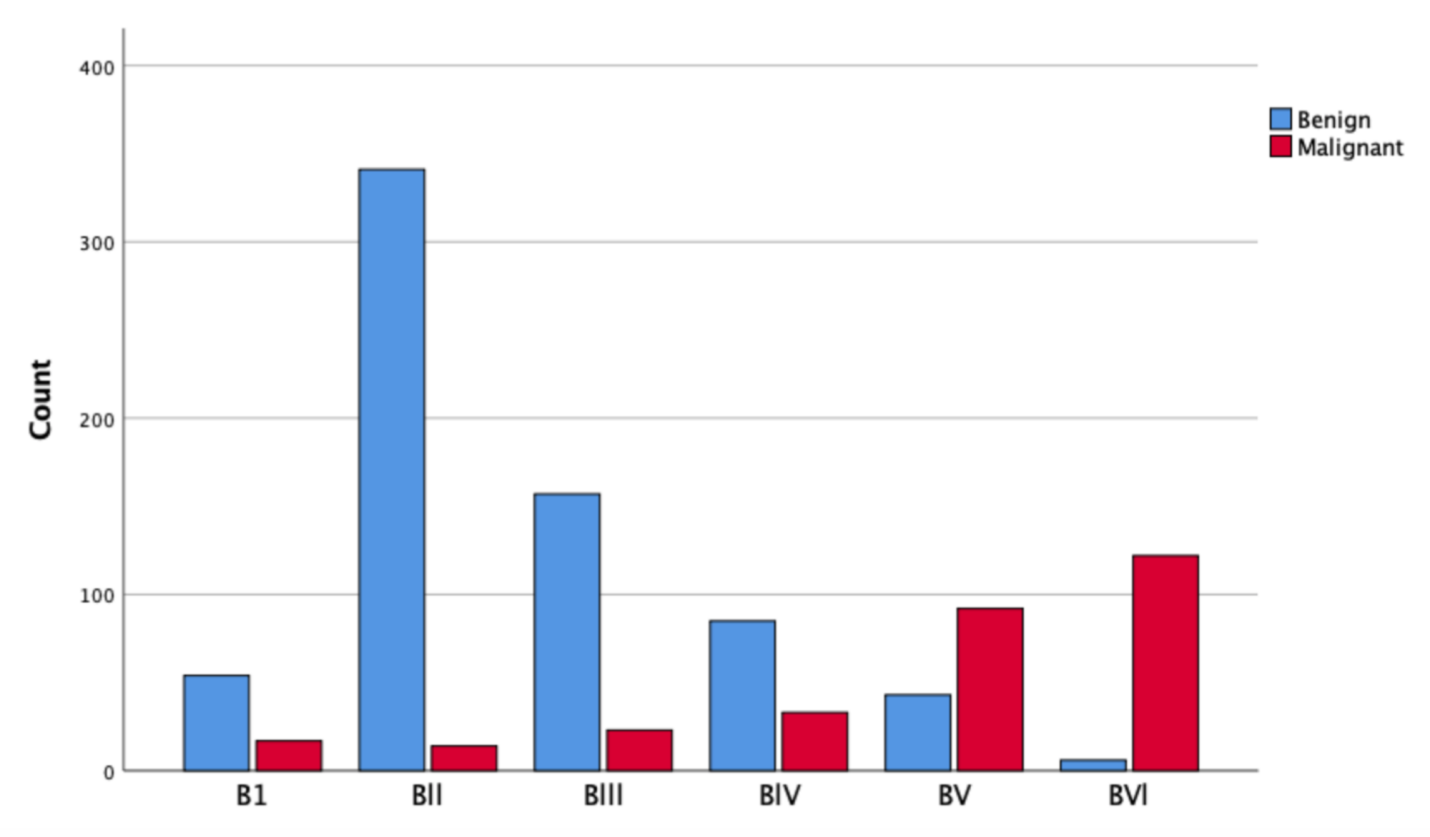
Correlation of fine needle aspiration (FNA) diagnosis based on Bethesda classification system with permanent histopathology results

The findings of the preoperative fine needle aspiration cytology (FNAC) showed that incidence of malignancy increases from B(I) to B(VI) with the highest malignancy incidence noted in B(VI) which is a good predictor of malignancy (p< 0.001) (Figure 1). 

A comparison between malignant and benign thyroid nodules is shown in Table 2; multiple factors were identified to be statistically significant variables. Malignant nodules were mostly prevalent in patients aged 45 and above (p<0.001). Benign thyroid nodules were found significantly higher in females, but the incidence of malignancy was higher in men (p<0.001). Regarding the number of nodules, the prevalence of malignancy in solitary thyroid nodules is higher than that of multinodular goiter (p<0.001). More patients with malignant nodules had a family history of thyroid cancer, and the majority of them had a normal thyroid function (p <0.001). Regarding nodular sizes, the cases with a nodular size of 1.0-1.9 cm were more prevalent among cancer patients than in benign cases (p<0.001). 

**Table 2:** Comparison between cancer and benign patients with thyroid nodules regarding demographic and clinical characteristics

Characteristics	Cancer patients (n=301) [n (%)]	Benign patients (n=686) [n (%)]	P
Age (years)			
<45	78 (25.9)	297 (43.3)	<0.001
≥45	223 (74.1)	389 (56.7)	
Gender			
Females	120 (39.9)	557 (81.2)	<0.001
Males	181 (60.1)	129 (18.8)	
Number of nodules			
Solitary	192 (63.8)	228 (33.2)	<0.001
Multiple	109 (36.2)	458 (66.8)	
Family history of thyroid cancer			
Yes	174 (57.8)	96 (14)	<0.001
No	127 (42.2)	590 (86)	
Thyroid function			
Hypothyroid	4 (1.3)	12 (1.7)	0.631
Euthyroid	295 (98)	632 (92.1)	<0.001
Hyperthyroid	2 (0.7)	42 (6.1)	0. 089
Size of nodules (cm)			
1.0‑1.9	195 (64.8)	96 (14)	<0.001
2.0‑2.9	53 (17.6)	266 (38.8)	
3.0‑3.9	32 (10.6)	182 (26.5)	
≥4	21 (7)	142 (20.7)	
Size of nodules (cm)			
1.0‑1.9	195 (64.8)	96 (14)	<0.001
≥2	106 (35.2)	590 (86)	

The postoperative histopathology results of 301 patients with malignant nodules are presented in Table 3. Papillary carcinoma was the most common type of malignant nodules (85.4%) followed by follicular carcinoma (8.3%), medullary carcinoma (3%), lymphoma (2%), and lastly anaplastic carcinoma (1.3%). 

**Table 3:** Pathological diagnosis of the studied patients with malignant thyroid nodules

Pathological diagnosis	Patients (n=301) [n (%)]
Papillary carcinoma	257 (85.4)
Follicular carcinoma	25 (8.3)
Medullary carcinoma	9 (3)
Lymphoma	6 (2)
Malignant anaplastic carcinoma	4 (1.3)

When the analysis of nodule size was compared with the type and distribution of thyroid malignancy, a significant relationship was detected. The majority of cancer cases, having a nodular size of 1.0-1.9 cm, were diagnosed as papillary carcinoma, and 61.9% of cancerous nodules ≥4 cm were follicular carcinomas. Also, an increase in the proportion of the other rare types of malignancy, such as medullary, anaplastic carcinoma, and thyroid lymphoma was noted with increasing nodule size (p<0.001) (Table 4).

**Table 4:** Frequency distributions of cancer types in relation to nodular size of cancer patients

Type of cancers	Size of thyroid nodules of cancer patients (n=301)			
	1-1.9 cm (n=195)	2-2.9 cm (n=53)	3-3.9 cm (n=32)	≥4 cm (n=21)
Papillary carcinoma	194 (99.5)	42 (79.2)	16 (50)	5 (23.8)
Follicular carcinoma	0	3 (5.7)	9 (28.1)	13 (61.9)
Medullary carcinoma	1 (0.5)	5 (9.4)	3 (9.4)	0
Lymphoma	0	1 (1.9)	2 (6.3)	3 (14.3)
Malignant anaplastic carcinoma	0	2 (3.8)	2 (6.3)	0

## Discussion

It is critical to detect and evaluate thyroid nodules for possible thyroid cancer because 5-15% of all thyroid nodules are histopathologically malignant [1]. In our study, we attempted to determine the relationship between nodule size and malignancy risk. We found out that thyroid cancer is more prevalent among patients ages 45 years and above as compared to patients aged below 45 years (p<0.001). In concurrence with our findings, Hughes et al. and El‐Gammal et al. reported similar results in their studies stating that thyroid cancer among patients aged greater than or equal to 45 years was statistically significant than those aged less than 45 years [7, 8]. Also, Reynolds et al. documented that thyroid cancer was more prevalent among the old age group than in the younger age group [9]. Chen et al. did not agree with the findings. Instead, they reported that thyroid cancer is common among people aged 45 years and below [10]. Witczak et al. reported that the age difference between patients with malignant diseases and those with benign diseases is not significant [11].

Based on our study, male patients had a higher incidence of malignancy than female patients. In agreement with our study, Paul et al. reported that the incidence of malignancy is higher in men [12]. Moreover, Li et al. reported that the incidence of thyroid cancer in male patients was significantly higher than in female patients (p=0.003) [13]. In contrast to our results, studies conducted by El‐Gammal et al. and Witczak et al. reported a high incidence of malignancy more in females than males [8, 11]. However, a study by Jaheen et al. found that the difference in the incidence of malignancy between men and women is not statistically significant [14].

Our study found that the highest malignancy risk was observed in nodules <2 cm and no increase in malignancy risk for nodules >2 cm. Thyroid nodules 1.0-1.9 cm in diameter provided baseline cancer risk for comparison (64.8% risk of cancer). The overall prevalence of cancer in nodules 2.0-2.9 cm was 17.6%; in nodules 3.0-3.9 cm it was 10.6%; and in nodules 4.0 cm it was 7%, presenting with statistically significant difference (p<0.001). However, the primary influence of this association was the high malignancy rate in nodules 1.0-1.9 cm. When comparing nodules 2.0-2.9 cm, 3.0-3.9 cm, or 4.0 cm, no difference in malignancy rate was demonstrated. This suggests a possible threshold effect. Hence, it’s worth suggesting that thyroid nodule size up to 2 cm is associated with an increased risk of thyroid cancer, but further growth beyond 2 cm no longer influences cancer risk. 

El‐Gammal et al. and Kamran et al. reported similar findings that a notable threshold effect is detected at ∼2.0 cm. However, further growth beyond 2 cm no longer impacts malignant risk [8, 15]. Moreover, Albuja‐Cruz et al. reported that there was no significant difference between the >4 cm group and the <4 cm group in terms of malignancy (p=0.91) [15]. Moreover, Megwalu et al. have studied nodules ≥4 cm and have not detected an association between nodule size and malignancy (p=0.7) [16]. In addition, Cavallo et al. reported that thyroid nodule size is inversely related to malignancy risk, as larger nodules have lower malignancy rates [17]. Also, McHenry et al. reported that nodule size does not appear to be an independent predictor of thyroid malignancy, suggesting that it should not be used in lieu of FNAB for therapeutic decision making [18]. Furthermore, Bohacek et al. suggested that there is no trend toward increased malignancy in larger nodules [19]. On the other hand, Kuru et al. found that the incidence of thyroid cancer was significantly higher in nodule size greater than or equal to 4 cm [20]. Carrillo et al. reported that FNAB results were highly inaccurate in nodule ≥4cm, 26 of 52 (50%) were reported as benign turned out to be malignant on final pathology [21]. Additionally, Shin et al. reported that large nodules have a higher pretest probability of malignancy [22]. Moreover, Smith‐Bindman et al. reported that the risk of malignancy is higher in nodules >2 cm compared with nodules under 1 cm [23]. Also, Hong et al. reported that the risk of malignancy differed according to the US pattern. In intermediate- and low-suspicion nodules, a large nodule size (≥ 3 cm) showed a higher malignancy risk than smaller nodules [24]. As seen, there are conflicting results from different studies. Hence, nodule size on its own is not a reliable variable for predicting malignancy, and deciding any medical interventions based on it would not be a correct approach.

Regarding the number of nodules, in our study, 987 study patients included 420 (42.6%) with a solitary thyroid nodule and 567 (57.4%) with multinodular goiter (MNG). The prevalence of thyroid cancer was statistically significant between solitary nodules and MNG (p<0.001). Out of the 567 patients with MNG, there were 458 patients with benign nodules and 109 patients with malignant nodules. On the contrary, out of 420 patients with solitary thyroid nodules, there were 192 patients with malignant nodules and 228 with benign nodules. Hence, we suggest that solitary thyroid nodules are a predictor of malignancy. In agreement with our study, Frates et al. reported that solitary nodules had a higher likelihood of malignancy than a non-solitary nodule (p <0.01) [25]. Furthermore, Li et al. reported that the incidence of thyroid cancer in patients with a single nodule was significantly higher than in patients with multiple nodules (p<0.001) [13]. Multinodular goiters have been known as a benign condition with a low risk of associated malignancy [26]. Nevertheless, Gandolfi et al. reported that MNG should no longer be considered an indicator of probable benign disease and should be assessed as if it were a solitary nodule [27]. Additionally, El‐Gammal et al. found that MNG were statistically significant for malignancy compared to solitary thyroid nodules [8]. 

Moreover, in our study, we found that the majority of patients with thyroid cancer had serum TSH concentration in a normal range, and it was statistically significant (p<0.001). In agreement with our findings, El‐Gammal et al. reported that all patients with thyroid cancer in their study had normal thyroid function [8]. In contrary, Li et al. reported that high serum TSH level is an independent risk factor of thyroid cancer [13].

When analysis of nodule size was compared with the type and distribution of thyroid malignancy, papillary carcinoma was predominant in nodular size of 1.0-1.9 cm, and follicular carcinoma was found significantly in nodule size ≥4 cm. In agreement with our study, Kamran et al. found that increasing nodule size was associated with a lower proportion of papillary carcinomas (p<0.01) [6]. However, El‐Gammal et al. reported that papillary carcinoma was predominant in larger sizes (≥2 cm) [8].

## Conclusions

To conclude, the findings of this study suggest that an increase in thyroid nodule size impacts cancer risk in a nonlinear fashion. A threshold is detected at 2 cm, and extension beyond 2 cm does not influence cancer risk. Nevertheless, the risk of follicular carcinomas and other rare thyroid malignancies increases as nodules enlarge.
